# NPC1L1 knockout protects against colitis-associated tumorigenesis in mice

**DOI:** 10.1186/s12885-015-1230-0

**Published:** 2015-03-27

**Authors:** Jianming He, Hyunsu Shin, Xing Wei, Anil Kumar G Kadegowda, Rui Chen, Sandy Krystal Xie

**Affiliations:** 1 Department of Oncology and Southwest Cancer Center, Southwest Hospital, Third Military Medical University, Chongqing, 400038 People’s Republic of China; 2 Department of Animal and Avian Science, University of Maryland, College Park, MD 20742 USA; 3 Department of Pathology, Chongqing Cancer Hospital, Chongqing, 400030 People’s Republic of China

**Keywords:** NPC1L1, Colorectal cancer, Tumorigenesis, Cholesterol, β-catenin, p53

## Abstract

**Background:**

Colorectal cancer is strongly associated with lipid metabolism. NPC1L1, a sterol transporter, plays a key role in modulating lipid homeostasis in vivo. Its inhibitor, ezetimibe, began to be used clinically to lower cholesterol and this caused the great debate on its role in causing carcinogenesis. Here we explored the role of NPC1L1 in colorectal tumorigenesis.

**Methods:**

Wild-type mice and NPC1L1^−/−^ (NPC1L1 knockout) mice were treated with azoxymethane (AOM)-dextran sodium sulfate (DSS) to induce colitis-associated colorectal tumorigenesis. Mice were sacrificed 10, 15, 18 or 20 weeks after AOM treatment, respectively. Colorectal tumors were counted and analyzed. Plasma lipid concentrations were measured using enzymatic reagent kit. Protein expression level was assayed by western blot.

**Results:**

NPC1L1^−/−^ mice significantly had fewer tumors than wild-type. The ratio of malignant/tumor in NPC1L1^−/−^ mice was significantly lower than in wild-type 20 weeks after AOM-DSS treatment. NPC1L1 was highly expressed in the small intestine of wild-type mice but its expression was undetectable in colorectal mucous membranes or tumors in either group. NPC1L1 knockout decreased plasma total cholesterol and phospholipid. NPC1L1^−/−^ mice had significant lower intestinal inflammation scores and expressed inflammatory markers p-c-Jun, p-ERK and Caspase-1 p20 lower than wild-type. NPC1L1 knockout also reduced lymphadenectasis what may be caused by inflammation. NPC1L1 knockout in mice decreased β-catenin in tumors and regulated TGF-β and p-gp in adjacent colons or tumors. There was not detectable change of p53 by NPC1L1 knockout.

**Conclusions:**

Our results provide the first evidence that NPC1L1 knockout protects against colitis-associated tumorigenesis. NPC1L1 knockout decreasing plasma lipid, especially cholesterol, to reduce inflammation and decreasing β-catenin, p-c-Jun and p-ERK may be involved in the mechanism.

**Electronic supplementary material:**

The online version of this article (doi:10.1186/s12885-015-1230-0) contains supplementary material, which is available to authorized users.

## Background

Cancer is a major public health problem worldwide, causing 1 in 4 deaths in the United States [1]. Colorectal cancer (CRC) ranks as the third highest incidence and mortality rates among men and women [1].

Evidence is accumulating that lipid metabolism is strongly associated with cancer, including CRC [1-4]. About 11% of CRC cases have been attributed to overweight and obesity in Europe. On the other hand, obesity is associated with a 30-70% increased risk of colon cancer in men [2]. High serum triglyceride (TG) and total cholesterol (TC), especially low-density lipoprotein cholesterol (LDL-C), are significantly and positively associated with cancer (including CRC) [1-4].

Serum lipid is modulated by many factors, including some genes, such as leptin, adipose triglyceride lipase and so on. Among these genes, *Niemann-Pick C1 like 1 (NPC1L1)* has been proven to be one of the most important sterol transporters [5]. It was reported that it plays a role in modulation of lipid homeostasis, including TG, phospholipid (PL), low-density lipoprotein, high-density lipoprotein, and most of all, cholesterol, in mice [6-8]. In clinic, *NPC1L1* genotype was found to be significantly associated with plasma lipid concentration, especially TC and LDL-C [9]. The NPC1L1 inhibitor, ezetimibe, began to be used in clinic to lower cholesterol and has caused the great debate on its role in cancer. Dr. Rossebo reported that using ezetimibe with simvastatin to lower serum lipid caused a significant increase of new cancer incidents [6]. Dr. Peto disputed that analyses of cancer data from several ezetimibe trials did not provide credible evidence of adverse effect on rates of cancer [7].

Here, we explored the role of NPC1L1 in colorectal tumorigenesis in vivo by using transgenic mice. Our results testify that NPC1L1 knockout in mice protects against colitis-associated tumorigenesis.

## Methods

### Animals and diets

NPC1L1^−/−^ mice were described previously [8,9]. Wild-type (WT) mice and NPC1L1^−/−^ mice were derived from NPC1L1^+/−^ mice with a pure C57BL/6 background and housed in a specific pathogen-free animal facility in plastic cages in a temperature controlled room (22°C) with a daylight cycle from 6 AM to 6 PM. Mice had free access to a standard laboratory rodent chow diet (Rodent NIH-07 22.5-5; Zeigler Bros Inc., Gardners, USA) and water. All animal procedures were approved by the Institutional Animal Care and Use Committee at University of Maryland Health Sciences.

### AOM-DSS induced colitis-associated tumorigenesis

AOM-DSS induced colorectal tumorigenesis was described previously [10,11]. Six to seven week-old, male mice were given a single intraperitoneal injection of 10 mg/kg body weight AOM (Sigma-aldrich, USA). Starting at a week after injection, animals received DSS (MP Biomedicals, Molecular weight was 36,000–50,000 Da., USA) for 7 days via free access to drinking water containing 2% DSS. The DSS water was replaced every day. Then, mice were fed with regular water and there was not any further treatment after DSS treatment.

Mice were sacrificed at different time points and colorectums were excised. Colorectums were opened longitudinally, flushed with ice-cold PBS and fixed in 10% formalin/PBS. Then macroscopic tumors were counted and measured with a caliper. Enlarged abdominal (from sciatic, lumbar, para-aortic to truncus coeliacus) lymph nodes were counted, collected and fixed in 10% formalin/PBS.

### Histological analysis

10% formalin/PBS fixed colorectums and enlarged lymph nodes were sent to Histoserv INC. (Germantown, MD, USA) for paraffin embedding and hematoxylin and eosin (H&E) staining. In brief, paraffin embedded samples were cut into 6 μM and stained with H&E. A pathologist counted the malignant tumors (including high grade intraepithelial neoplasia and adenocarcinoma) and benign tumors in each slide. The malignant/tumor ratio of each slide was calculated and the average ratio of each group was presented. Inflammation was scored on a 0–4 scale (0, normal mucosa; 1, minimal inflammation (occasional scattered granulocytes and leukocytes); 2, mild inflammation (scattered granulocytes with occasional mild infiltrates); 3, moderate inflammation (scattered granulocytes with patchy moderate infiltrates); and 4, severe inflammation (multiple extensive areas with abundant granulocytes and marked infiltrates))[12].

### Plasma lipid concentrations measurement

Mice were sacrificed with isoflurane. Blood was collected by inferior vena cava injection in an anticoagulant tube and the tube was centrifuged at 800 g for 15 min at 4°C. Supernatant was collected as plasma. Plasma free cholesterol (FC), PL were measured using enzymatic reagent kit from Wako Diagnostics (USA), TC was measured using enzymatic reagent kit from Pointe Scientific (USA). TG was measured using enzymatic reagent kit from Sigma-Aldrich (USA).

### Western blot analysis

To obtain total protein, multiple tissues in RIPA Buffer (50 mM Tris base, 150 mM NaCl, 1% Nonidet P-40, 0.25% Na-deoxycholate, 1 mM EDTA) with protease inhibitors (1 mM PMSF, 5 μg/ml leupeptin, 2 μg/ml pepstatin, 4 μg/ml aprotinin) and phosphatase inhibitors (10 mM NaF, 1 mM Na_3_VO_4_, 10 mM β-Glycerophosphate disodium salt pentahydrate) were homogenized for 60 seconds on ice. Tissue homogenates were centrifuged at 13,000 rpm for 20 minutes at 4°C and the supernatant was collected as total cell protein.

To obtain membrane protein, colon mucous membrane or colorectal tumors in Membrane Buffer (20 mM Tris–HCl pH7.5, 2 mM MgCl_2_, 250 mM Sucrose) with protease inhibitors and phosphatase inhibitors were homogenized for 60 seconds on ice. Tissue homogenates were centrifuged at 2,000 rpm for 10 minutes at 4°C. The supernatant was transferred to a new tube and the tube was centrifuged at 110,000 g for 30 min at 4°C. The supernatant was removed and left the tube upside down to drain for 1 minute. Then, the pellet was resuspended in Sample Buffer (50 mM Tris–HCl pH8.0, 80 mM NaCl, 4 mM CaCl_2_, 1%(v/v) Triton X-100) with protease inhibitors and phosphatase inhibitors. This was membrane protein.

Protein was resolved by SDS/PAGE and blotted on Nitrocellulose Membranes (Bio-Rad, USA) as previously described[13]. Nitrocellulose Membranes were incubated with specific primary antibodies overnight. After incubating with secondary antibodies, immunoreactive proteins were visualized by the Enhanced Chemiluminescnet Substrate (Pierce, USA).

E-cadherin antibody, β-catenin antibody, TGF-β antibody, p-c-Jun (Ser63) antibody, p-Erk1/2 (Thr202/Tyr204) antibody, β-actin antibody, α-tubulin antibody, HRP-linked secondary antibody were from Cell signal Technology (USA). Caspase-1 p20 antibody, p-gp antibody, p53 antibody was from Santa Cruz Biotechnology, Inc. (USA).

### Statistics

The data shown represent the mean ± standard error. Statistical differences between groups were analyzed by Student’s t-test, one-way ANOVA or chi-square test. *P* < 0.05 was considered statistically significant.

## Results

### Azoxymethane (AOM)-dextran sodium sulfate (DSS) treatment induces tumors only in colorectum

**Figure 1:**
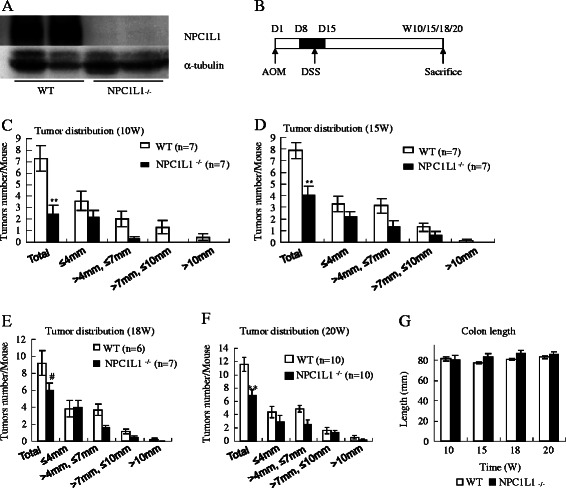
**NPC1L1 knockout protects mice against colitis-associated tumorigenesis.(A)** NPC1L1 protein expression was absent in the small intestine 2/5 of NPC1L1^−/−^ mice by western blot analysis. **(B)** Colorectal tumorigenesis was induced by injection of the procarcinogen AOM followed by one round of DSS exposure to elicit colitis. WT mice and NPC1L1^−/−^ mice were sacrificed at 10 weeks (10 W), 15 weeks (15 W), 18 weeks (15 W) or 20 weeks (20 W) after AOM injection, respectively. **(C-F)** Tumors were measured and divided into four groups according to the maximum diameter (Group 1: ≤4 mm; Group 2: >4 mm but ≤7 mm; Group 3: >7 mm but ≤10 mm; Group 4: >10 mm). Tumor number of each group and total tumor number distribution was diagrammed. With increased time post-injection, tumors increased in numbers and size. NPC1L1^−/−^ mice had fewer tumors than WT mice. **p < 0.01, #*p* = 0.095 compared to WT. **(G)** When mice were sacrificed, colorectums (The cecum was not included.) were measured. There was not any significant difference in colon length between NPC1L1^−/−^ mice and WT mice at any time points.

**Figure 2:**
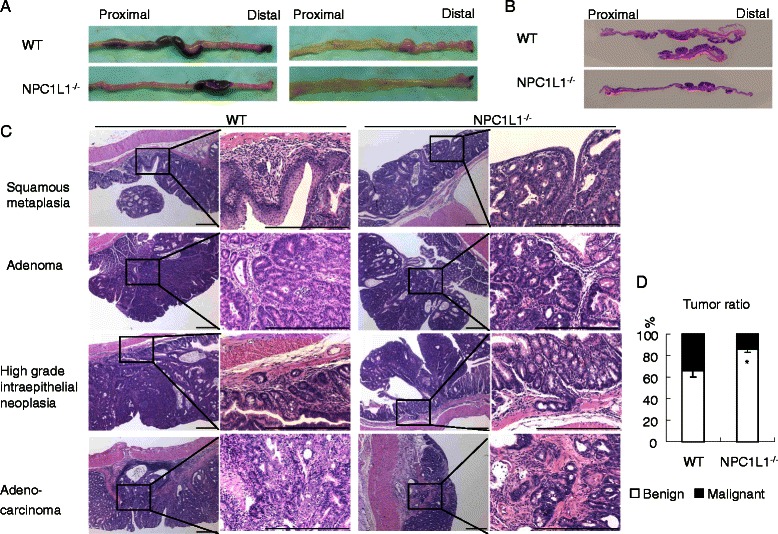
**Pathological changes.(A)** Colons were excised 10 weeks after AOM injection (left) and were longitudinally opened (right). **(B)** Colons were excised 20 weeks after AOM injection and were stained with H&E. **(C)** Squamous metaplasia, adenoma, high grade intraepithelial neoplasia and adenocarcinoma were found in WT group and NPC1L1^−/−^ group. (Scale bars represent 400 μm.) **(D)** NPC1L1^−/−^ mice had a lower ratio of malignant tumor/tumor than WT mice. *p < 0.05.

To explore colorectal tumorigenesis in vivo, the widely used AOM-DSS model of colitis-associated colorectal tumorigenesis was employed [10,11]. Mice were sacrificed 10, 15, 18 or 20 weeks after AOM injection, respectively (Figure 1B). Macroscopic tumors were counted and measured. The detail information was summarized in Figures 1, 2 and Table 1. Tumors were mainly distributed on the distal part of colorectum, with more distal tumors increasing in density (Figures 2A,B). Tumors were not found in cecum or small intestine. With increased time post-injection, tumors increased in numbers and size (Figures 1C-F).

**Table 1:** **Number of mice with tumors**

		≥10 mm	≥7 mm	≥4 mm	With tumor	Total
10 W	WT	2	4	6	7	7
	NPC1L1^−/−^	0	0	2	6	7
15 W	WT	1	6	7	7	7
	NPC1L1^−/−^	0	2	5	7	7
18 W	WT	1	5	6	6	6
	NPC1L1^−/−^	0	3	7	7	7
20 W	WT	5	9	10	10	10
	NPC1L1^−/−^	2	8	10	10	10

The liver, lung, spleen and kidneys of each mouse were examined and neither tumor nor metastatic cancer was found (Additional file 1: Figure S1E).

### NPC1L1 knockout significantly decreases colitis-associated tumorigenesis

The role of NPC1L1 in tumorigenesis was explored by using genetic knockout mice. Western blot was used to confirm that NPC1L1 was knockout in mice (Figures 1A). Sex- and age-matched WT mice and NPC1L1^−/−^ (NPC1L1 knockout) mice were administrated with AOM-DSS. NPC1L1^−/−^ mice significantly had fewer tumors than WT mice (Figures 1C-F). NPC1L1^−/−^ mice also had smaller tumors but without a significance (Figures 1C-F, Table 1). Tumor numbers per mouse of WT at 10 W, 15 W, 18 W and 20 W were 7.29 ± 1.11, 7.86 ± 0.67, 9.17 ± 1.46 and 11.60 ± 1.05, respectively. Those of NPC1L1^−/−^ were 2.43 ± 0.78, 4.00 ± 0.79, 6.00 ± 0.87 and 6.90 ± 0.89, respectively.

The difference of susceptibility to tumorigenesis between WT mice and NPC1L1^−/−^ mice does not appear to be caused by colon length because no significant difference in colon length between the two was detected (Figure 1G).

Mice at 12 weeks and 24 months of age (including WT and NPC1L1^−/−^) without any treatment were sacrificed and did not show tumors in the colorectum, cecum, small intestine, lung, liver, spleen or kidneys. This suggests NPC1L1 knockout alone should not cause tumorigenesis in those organs within 24 months.

### NPC1L1^−/−^ mice have a lower ratio of malignant tumor/tumor than WT

Eight colorectums of 20 W mice from each group were used to do H&E staining. All slides were examined by a pathologist. Squamous metaplasia, infiltration of inflammatory cells, adenoma, high grade intraepithelial neoplasia and adenocarcinoma were found in colorectums of both WT group and NPC1L1^−/−^ group (Figure 2C). NPC1L1^−/−^ mice had a significant lower ratio of malignant tumor/tumor than WT (*p* < 0.05) (Figure 2D). Malignant tumor/tumor ratio of WT was 33.9 ± 6.1% while that of NPC1L1^−/−^ was 14.2 ± 2.8%.

### NPC1L1 in mice colorectal mucous membranes or colorectal tumors is undetectable by western blot

**Figure 3:**
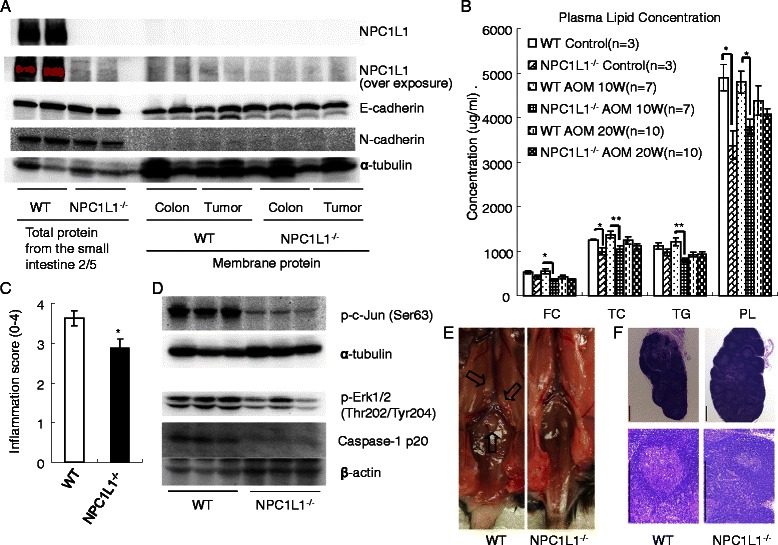
**NPC1L1 knockout decreasing plasma lipid and reducing inflammatory sensitivity may be involved in the mechanism.(A)** Western blot. Total proteins from small intestines and membrane proteins from adjacent colon mucous membranes or tumors were blotted. NPC1L1 in mice colorectal mucous membranes or colorectal tumors was undetectable by western blot. Membrane protein loading control α-tubulin and membrane protein E-cadherin were easily detected in tumors or adjacent colorectal mucous membranes. N-cadherin was easily detected in small intestine tissues while it was undetectable in tumors or adjacent colorectal mucous membranes. **(B)** Plasma samples from 12 W old mice without any treatment (control), 10 W or 20 W after AOM injection were assayed for FC, TC, TG and PL. *p < 0.05, **p < 0.01. **(C)** The intestinal inflammation scores of NPC1L1−/− mice were significantly lower than those of WT mice. *p < 0.05. **(D)** Western blot. NPC1L1 ablation decreased inflammatory markers p-c-Jun, p-ERK and Caspase-1 p20 in tumors. **(E)** Fresh enlarged (sciatic and lumbar) lymph nodes. Mice were sacrificed 20 weeks after AOM injection. **(F)** H&E staining of enlarged lymph nodes showed that metastasis was not found. (Scale bars represent 400 μm).

It was reported that NPC1L1 mRNA expression in both mice colon and human colon was very low [8,14]. Western blot was employed to assay NPC1L1 protein in intestine and colorectal tumors. At first, total proteins from adjacent colorectal mucous membranes and tumors were used and NPC1L1 signal was not detectable. Because NPC1L1 is mainly expressed in cell membrane [14,15], membrane proteins from adjacent colorectal mucous membranes and tumors were blotted at last. Small intestines were divided into 5 segments and total protein from the second segment was used as a control. As shown in Figure 3A, NPC1L1 was highly expressed in the jejunum of WT mice and was not detectable in that of the NPC1L1^−/−^ mice. NPC1L1 signal was not detectable in tumors or adjacent colorectal mucous membranes in either group. To confirm this result, western blot membrane was over exposed and the result was the same.

To confirm the quality of membrane protein, membrane protein loading control α-tubulin and membrane protein E-cadherin were both detected on the same membrane. As shown in Figure 3A, α-tubulin and E-cadherin were easily detected by western blot. N-cadherin was also assayed. It was easily detected in total protein of the jejunum and was undetectable in tumors or adjacent colorectal mucous membranes (Figure 3A).

### NPC1L1 knockout decreases plasma lipid, especially TC

NPC1L1 plays a key role in modulating lipid homeostasis in vivo and serum lipid are reported to promote colorectal tumorigenesis [2-4,8,15,16]. Therefore, plasma lipids were assayed (Figure 3B).

Plasma TC concentration of NPC1L1^−/−^, as expected, was much lower than that of WT. TC concentration of NPC1L1^−/−^ group without any treatment was 992 ± 82 ug/ml while that of WT group was 1253 ± 5 ug/ml (*p* < 0.05). At week 10, plasma TC concentration of NPC1L1^−/−^ group (1047 ± 75 ug/ml) was significantly lower than that of WT group (1377 ± 68 ug/ml) (*p* < 0.01). At week 20, NPC1L1^−/−^ mice had a lower concentration of TC (1113 ± 59 ug/ml) compared to WT mice (1239 ± 91 ug/ml), but without significance (*p* > 0.05).

NPC1L1^−/−^ mice had a lower FC than WT mice only at week 10, where there was a significant difference (*p* < 0.05). Plasma FC concentrations of NPC1L1^−/−^ mice without treatment, at week 10 and week 20 were 416 ± 41 ug/ml, 359 ± 35 ug/ml and 373 ± 19 ug/ml, respectively. FC concentrations of WT mice were 534 ± 35 ug/ml, 547 ± 59 ug/ml and 422 ± 39 ug/ml, respectively.

Untreated NPC1L1^−/−^ mice and mice treated at week 20 had a similar TG to WT mice (*p* > 0.05). TG concentrations of WT mice without treatment, at week 20 were 1123 ± 79 ug/ml and 921 ± 49 ug/ml, respectively. TG concentrations of NPC1L1^−/−^ mice were 976 ± 69 ug/ml and 932 ± 64 ug/ml, respectively. At week 10, NPC1L1^−/−^ mice had a lower TG (799 ± 48 ug/ml) than WT mice (1210 ± 95 ug/ml) (*p* < 0.01).

NPC1L1^−/−^ mice had lower plasma PL than WT mice. The plasma PL concentration of NPC1L1^−/−^ group without any treatment was 3379 ± 322 ug/ml while that of WT mice was 4892 ± 294 ug/ml (*p* < 0.05). At week 10, plasma PL concentration of NPC1L1^−/−^ group was 3798 ± 163 ug/ml while plasma PL concentration of WT group was 4801 ± 243 ug/ml (*p* < 0.01). At week 20, plasma PL concentration of NPC1L1^−/−^ group was 4081 ± 116 ug/ml while that of WT group was 4380 ± 327 ug/ml (*p* > 0.05).

### NPC1L1 knockout reduces sensitivity to inflammatory agent

It is reported that lipid promotes colitis-associated tumorigenesis through inflammation in mice [16]. Degrees of intestinal inflammation were evaluated for each group. Intestinal inflammation scores were 3.6 ± 0.2 in WT and 2.9 ± 0.2 in NPC1L1^−/−^, respectively (P < 0.05) (Figure 3C).

Protein p-c-Jun, p-ERK and Caspase-1 are thought as inflammatory markers and are involved in carcinogenesis [17-22]. Therefore, p-c-Jun, p-ERK and Caspase-1 p20 protein expression in tumors were assayed by western blot. NPC1L1 knockout significantly and dramatically decreased p-c-Jun. Both p-ERK and Caspase-1 p20 were also decreased (Figure 3D).

### NPC1L1 knockout decreases lymphadenectasis

Inflammation and cancer metastasis are two main causes in lymphadenectasis [23,24]. In this study, enlarged abdominal lymph nodes were examined (Figures 3E,F and Table 2). Lymph nodes H&E staining showed that no metastatic cancer was found (Figure 3F). This led to the hypothesis that enlarged abdominal lymph nodes may be caused by inflammation. The number of enlarged abdominal lymph nodes in WT mice was higher than that found in NPC1L1^−/−^ mice (Figure 3E and Table 2). At week 10, all seven WT mice had enlarged abdominal lymph node and 5 mice had more than one. Three of the 7 NPC1L1^−/−^ mice had enlarged lymph nodes and none of them had more than one. The WT group had 1.9 ± 0.3 enlarged lymph nodes per mouse and the NPC1L1^−/−^ group had 0.4 ± 0.2 (*p* < 0.01). At week 20, all WT mice had enlarged lymph node and 9 of the 10 mice had more than one while 8 of the 10 NPC1L1^−/−^ mice had enlarged lymph nodes and only 2 mice had more than one. The WT group had 2.6 ± 0.3 enlarged lymph nodes while the NPC1L1^−/−^ group only had 1.0 ± 0.2 (*p* < 0.01).

**Table 2:** **Number of mice with enlarged lymph nodes**

Number of enlarged lymph Nodes		4	3	2	1	0	Total	*P*value
**10 W**	WT	0	1	4	2	0	7	0.001
	NPC1L1^−/−^	0	0	0	3	4	7	
**15 W**	WT	0	0	5	2	0	7	0.08
	NPC1L1^−/−^	0	1	0	3	3	7	
**18 W**	WT	1	1	3	1	0	6	0.08
	NPC1L1^−/−^	0	1	1	4	1	7	
**20 W**	WT	2	3	4	1	0	10	0.004
	NPC1L1^−/−^	0	0	2	6	2	10	

NPC1L1^−/−^ (total): *p* = 0.0001 vs WT (total).

### The effect of NPC1L1 knockout on β-catenin/p53/TGF-β/p-gp in colitis-associated tumorigenesis was evaluated

To explore the possible involved pathways, western blot was employed to assay β-catenin/p53/TGF-β/p-gp. The specific protein expression level in adjacent colons and tumors was measured on the same membrane.

**Figure 4:**
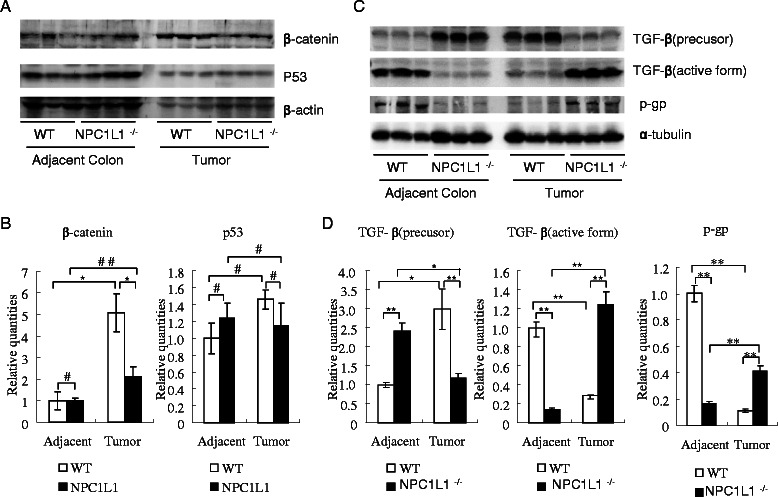
**Proteins (p53, β-catenin, TGF-β and p-gp) associated with tumorigenesis were assayed by western blot.(A, B)** Protein β-catenin and p53 of adjacent colons and tumors were assayed by western blot. Relative quantities of β-catenin and p53 were analyzed and normalized to actin, respectively. **(C, D)** TGF-β and p-gp of adjacent colons and tumors were assayed by western blot. Relative quantities of TGF-β and p-gp were analyzed and normalized to α-tubulin, respectively. *p < 0.05, **p < 0.01, #*p* >0.05, ##*p* =0.08.

β-catenin was much higher in tumors than in adjacent colons in both groups. NPC1L1 knockout significantly decreased β-catenin expression level in tumors but no changes were detected in adjacent colons (Figures 4A,B).

NPC1L1 knockout in mice didn’t detectably change p53 expression in tumors or adjacent colons (Figures 4A,B).

Interestingly, NPC1L1 knockout dramatically increased active TGF-β in tumors but dramatically decreased it in adjacent colons. On the contrary, NPC1L1 knockout decreased TGF-β precursor in tumors and increased it in adjacent colons (Figures 4 C, D).

NPC1L1 knockout in mice dramatically increased p-gp in tumors and the opposite in adjacent colons (Figures 4C,D).

## Discussion

NPC1L1 protein, a protein of more than 1,300 amino acids, has 13 transmembrane domains and 5 of them constitute a sterol-sensing domain [5,25]. Its major function is a sterol transporter [5,8,15]. The NPC1L1 inhibitor, ezetimibe, began to be used to lower serum cholesterol in clinic but this has caused the great debate [6,7]. Biological scientists and clinical doctors raised the concern about its role in cancer recently.

Here, we testified that NPC1L1^−/−^ mice were resistant to colitis-associated tumorigenesis. At different time points, NPC1L1^−/−^ mice consistently had much fewer colorectal tumors than WT mice (Figures 1C-G). The ratio of malignant tumor/tumor in NPC1L1^−/−^ at week 20 was also significantly lower than in WT (Figure 2D).

To explore clues to possible mechanisms of NPC1L1 knockout protecting mice against colitis-associated tumorigenesis, NPC1L1 protein in colorectal mucous membranes and tumors was assayed. Western blot showed that NPC1L1 protein level was high in small intestines while it was undetectable in colorectal mucous membranes or colorectal tumors (Figure 3A). It is reported that NPC1L1 mRNA was highly expressed in liver and small intestines of humans and only in small intestines of mice [8,14]. In the colon, its mRNA is very low [8,14]. Based on these results, NPC1L1 knockout reducing colitis-associated tumorigenesis is unlikely due to NPC1L1 ablation in colorectal mucous membranes.

NPC1L1 major function is a sterol transporter in small intestines and livers to modulate lipid homeostasis [8,15]. Plasma lipid, especially cholesterol, is strongly and positively associated with colorectal cancer and promotes colitis-associated tumorigenesis in mice [1-4,16]. Therefore, plasma lipid was assayed. NPC1L1 knockout significantly decreased plasma lipid, especially cholesterol (Figure 3B). Hence, plasma lipid, especially cholesterol, may be involved in the mechanism of NPC1L1 knockout reducing colitis-associated tumorigenesis.

Lipid, especially cholesterol, promoted colitis-associated tumorigenesis through inflammation [16]. To explore whether inflammation was involved in NPC1L1 knockout reducing colitis-associated tumorigenesis, the intestinal inflammation was scored. NPC1L1 knockout significantly decreased intestinal inflammation scores (Figure 3C). It significantly decreased p-c-Jun, p-ERK and caspase-1 p20, three inflammatory markers [17-20], in colorectal tumors (Figure 3D). Those suggested that NPC1L1 knockout reduced sensitivity to inflammatory agents. Therefore, NPC1L1 knockout decreasing plasma lipid, especially cholesterol, to reduce inflammation may be involved in the mechanism.

Lymphadenectasis also indicates inflammatory activity, somehow, because inflammation and cancer are main causes of lymphadenectasis [10,23,24]. H&E staining showed that none of enlarged lymph nodes were caused by metastatic cancer (Figure 3F). Therefore, NPC1L1^−/−^ mice having fewer enlarged lymph nodes (Figure 3E, Table 2) also suggests NPC1L1^−/−^ mice may be hyposensitive to inflammatory agent.

To explore clues to the detail mechanisms of NPC1L1 knockout reducing colitis-associated tumorigenesis, β-catenin, p-c-Jun, p-ERK, TGF-β, p-gp and p53 were assayed.

NPC1L1 knockout significantly decreased β-catenin, p-c-Jun and p-ERK in tumors. Increasing β-catenin, c-Jun phosphorylation and ERK activation were reported to promote colon tumorigenesis [10,11,21,22]. It is logical to consider that β-catenin, p-c-Jun and p-ERK may be involved in the mechanism of NPC1L1 knockout protecting mice from colitis-associated tumorigenesis.

TGF-β and p-gp also play a positive role during intestinal tumorigenesis [26,27]. Interestingly, NPC1L1 knockout dramatically increased active TGF-β and p-gp in tumor but dramatically decreased them in adjacent colon. On the contrary, NPC1L1 knockout decreased TGF-β precursor in tumor and increased it in adjacent colon (Figure 4B). It seems that TGF-β and p-gp might play complicated roles in mechanisms.

Protein p53, the most famous tumor suppressor protein, is involved in colorectal tumorigenesis and lipid metabolism [28,29]. Beyond our expectation, NPC1L1 knockout did not significantly change its expression either in adjacent colon or in tumor.

## Conclusions

NPC1L1 knockout in mice protects mice against colitis-associate colorectal tumorigenesis. This indicates NPC1L1 inhibitor might be used to protect against CRC as well as to lower cholesterol. Since *NPC1L1* genotype is different amongst people [30], our results may also contribute to explain differences in colorectal cancer susceptibility amongst people. NPC1L1 protein was undetectable in colorectal mucous membranes or tumors. NPC1L1 knockout decreased plasma lipid, especially cholesterol and reduced sensitivity to inflammatory agents. It is reported that lipid, especially cholesterol, promoted colitis-associated tumorigenesis through inflammation [16]. Therefore, NPC1L1 knockout decreasing plasma lipid, especially cholesterol, to reduce inflammation may be involved in the mechanism. NPC1L1 knockout decreasing β-catenin, p-c-Jun and p-ERK may also be involved in the mechanism. Further experiments are needed to clarify the complicated mechanism.

## Additional file

Additional file 1: Figure S1. Clinic parameters. **(A)** Body weight of WT began to decrease earlier than that of NPC1L1^-/-^. (When mice were injected with AOM, they were weighed and this body weight was regarded as original body weight). **(B)** Epididymal fat (EPI) weight/body weight ratio of WT began to decrease earlier than that of NPC1L1^-/-^. (**p*<0.05, #*p*>0.05). These suggested WT might have cachexia what might be caused by tumors earlier than NPC1L1^-/-^. **(C)** WT mice had higher ratio of liver weight/body weight than NPC1L1^-/-^ mice as we previously described (#*p*=0.076, **p*<0.05, ***p*<0.01 compared to WT). **(D)** WT mice had heavier spleen than NPC1L1^-/-^ mice and this might be due to anemia caused by DSS-induced colitis or tumor (#:*p*=0.060, ##*p*=0.079, **p*<0.05, ***p*<0.01 compared to WT). This suggested that WT might have more tumors and/or more serous inflammation than NPC1L1^-/-^. **(E)** Spleen, lung and kidney. Tumor wasn’t found either in WT group or in NPC1L1^-/-^ group.

## Abbreviations

AOM: Azoxymethane
CRC: Colorectal cancer
DSS: Dextran sodium sulfate
EPI: Epididymal fat
FC: Free Cholesterol
H&E: Hematoxylin and eosin
LDL-C: Low-density lipoprotein cholesterol
NPC1L1: Niemann-Pick C1 like 1
PL: Phospholipid
TC: Total cholesterol
TG: Triglyceride
WT: Wild-type
