# Supplementary material for: NPC1L1 knockout protects against colitis-associated tumorigenesis in mice
Source: BMC Cancer. 2015 Mar 27;15:189. doi: 10.1186/s12885-015-1230-0 (PMC4378275; doi:10.1186/s12885-015-1230-0)
Supplement: Additional file 1: Figure S1. — Clinic parameters. (A) Body weight of WT began to decrease earlier than that of NPC1L1-/-. (When mice were injected with AOM, they were weighed and this body weight was regarded as original body weight). (B) Epididymal fat (EPI) weight/body weight ratio of WT began to decrease earlier than that of NPC1L1-/-. (*p<0.05, #p>0.05). These suggested WT might have cachexia what might be caused by tumors earlier than NPC1L1-/-. (C) WT mice had higher ratio of liver weight/body weight than NPC1L1-/- mice as we previously described (#p=0.076, *p<0.05, **p<0.01 compared to WT). (D) WT mice had heavier spleen than NPC1L1-/- mice and this might be due to anemia caused by DSS-induced colitis or tumor (#:p=0.060, ##p=0.079, *p<0.05, **p<0.01 compared to WT). This suggested that WT might have more tumors and/or more serous inflammation than NPC1L1-/-. (E) Spleen, lung and kidney. Tumor wasn’t found either in WT group or in NPC1L1-/- group. [file 12885_2015_1230_MOESM1_ESM.ppt]

## Slide 1
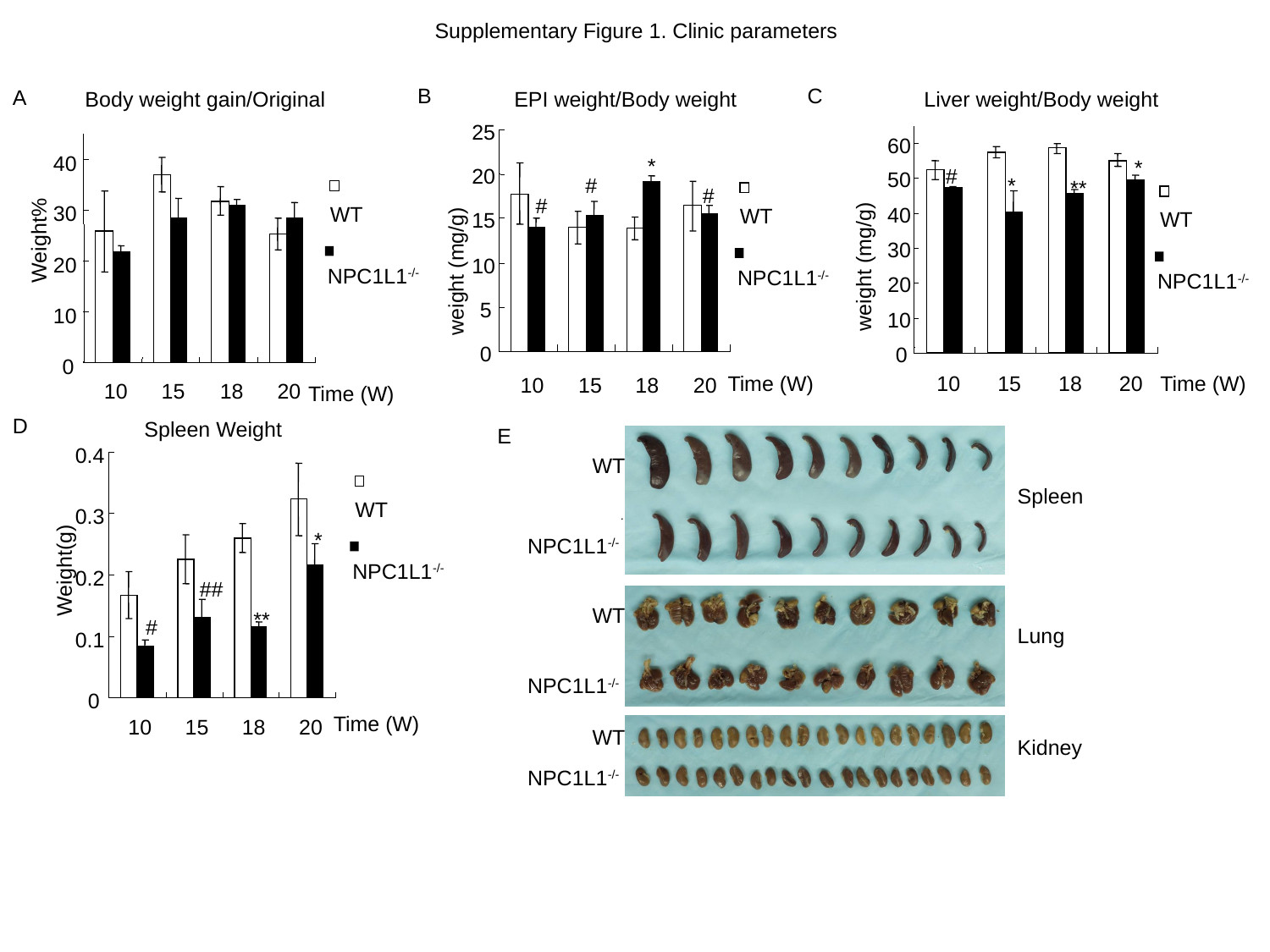

# Supplementary Figure 1. Clinic parameters
B
C
A
Body weight gain/Original
EPI weight/Body weight
Liver weight/Body weight
25
60
*
*
40
#
20
#
*
50
**
#
#
30
WT
40
WT
WT
15
Weight%
30
weight (mg/g)
20
10
weight (mg/g)
NPC1L1-/-
NPC1L1-/-
NPC1L1-/-
20
5
10
10
0
0
0
10
15
18
20
Time (W)
Time (W)
10
15
18
20
10
15
18
20
Time (W)
D
Spleen Weight
E
0.4
WT
Spleen
WT
0.3
*
NPC1L1-/-
NPC1L1-/-
Weight(g)
0.2
##
WT
**
#
Lung
0.1
NPC1L1-/-
0
Time (W)
10
15
18
20
WT
Kidney
NPC1L1-/-
